# Remote assessment of cognition in Parkinson’s disease and Cerebellar Ataxia: the MoCA test in English and Hebrew

**DOI:** 10.3389/fnhum.2023.1325215

**Published:** 2024-01-08

**Authors:** Sharon Binoy, Leila Montaser-Kouhsari, Penina Ponger, William Saban

**Affiliations:** ^1^Center for Accessible Neuropsychology and Sagol School of Neuroscience, Tel Aviv University, Tel Aviv-Yafo, Israel; ^2^Department of Occupational Therapy, Faculty of Medicine, Tel Aviv University, Tel Aviv-Yafo, Israel; ^3^Loyola Stritch School of Medicine, Chicago, IL, United States; ^4^Department of Neurology and Neurological Sciences, Stanford University, Stanford, CA, United States; ^5^Movement Disorders Division, Department of Neurology, Tel Aviv Sourasky Medical Center, Tel Aviv-Yafo, Israel

**Keywords:** online, neuropsychological testing, MoCA, Parkinson’s, ataxia, basal ganglia, cerebellum, Hebrew

## Abstract

There is a critical need for accessible neuropsychological testing for basic research and translational studies worldwide. Traditional in-person neuropsychological studies are inherently difficult to conduct because testing requires the recruitment and participation of individuals with neurological conditions. Consequently, studies are often based on small sample sizes, are highly time-consuming, and lack diversity. To address these challenges, in the last decade, the utilization of remote testing platforms has demonstrated promising results regarding the feasibility and efficiency of collecting patient data online. Herein, we tested the validity and generalizability of remote administration of the Montreal Cognitive Assessment (MoCA) test. We administered the MoCA to English and Hebrew speakers from three different populations: Parkinson’s disease, Cerebellar Ataxia, and healthy controls via video conferencing. First, we found that the online MoCA scores do not differ from traditional in-person studies, demonstrating convergent validity. Second, the MoCA scores of both our online patient groups were lower than controls, demonstrating construct validity. Third, we did not find differences between the two language versions of the remote MoCA, supporting its generalizability to different languages and the efficiency of collecting binational data (USA and Israel). Given these results, future studies can utilize the remote MoCA, and potentially other remote neuropsychological tests to collect data more efficiently across multiple different patient populations, language versions, and nations.

## 1 Introduction

Neuropsychological testing is essential for understanding cognitive processes and brain functionality across various patient groups, languages, and countries. Neuropsychological research provides insight into how different areas of the brain function, allowing for neuroanatomical localization and network level understanding of different brain regions (Grahn et al., 2009; Zald and Andreotti, 2010; O’Halloran et al., 2012). By testing individuals with brain disorders, we have advanced our understanding of brain-behavior relationships and gained a more detailed understanding of cognition (Lezak, 2000). In cognitive research, there is often a selection bias, focusing mainly on cortical function (Parvizi, 2009; Janacsek et al., 2022; Saban and Gabay, 2023). As a result, the role of subcortical regions is often overlooked (Saban et al., 2018a,b, 2021; Soloveichick et al., 2021).

However, studying patients with subcortical brain pathologies can help us understand the role of these regions in cognition (Rossetti et al., 2011; Malek-Ahmadi et al., 2018; Saban and Ivry, 2021; Saban and Gabay, 2023). For instance, neuropsychological testing of people with Parkinson’s disease (PD) provides insights into the function of the basal ganglia (BG) (Orozco et al., 2020). Similarly, studying individuals with Cerebellar Ataxia (CA) helps us understand the function of the cerebellum (Saban and Gabay, 2023).

Parkinson’s disease and CA are neurodegenerative disorders that affect the central nervous system, leading to profound impacts on motor processes. In PD, the loss of dopamine-producing neurons in the substantia nigra results in motor symptoms, such as tremors and rigidity. However, cognitive impairment is also a manifestation of PD, with deficits in executive function, attention, and memory (Kandiah et al., 2014; Weintraub et al., 2015; Moustafa et al., 2016). CA leads to difficulty in coordination, balance, and fine motor control. Cognitive impairment in CA is also expected as the disease progresses, especially in attention and executive functions (Fancellu et al., 2013; Tran et al., 2020; Malek et al., 2022). Motor function is frequently evaluated via the United Parkinson’s disease Rating Scale (UPDRS, Goetz, 2003) in PD, and in CA via the Scale for Assessment and Rating of Ataxia (SARA, Schmitz-Hübsch et al., 2006). Testing the cognitive profiles of both the PD and CA populations can allow researchers to better understand how subcortical brain structures relate to human cognitive abilities. The Montreal Cognitive Assessment (MoCA; Nasreddine et al., 2005), a globally recognized test, is commonly used by healthcare providers and researchers to screen and assess cognition in a wide variety of neurological diseases, including both PD and CA.

While testing cognition in PD and CA is crucial for understanding cognitive processes and the human brain, recruitment and testing of these individuals in person is challenging. This is partly due to the rarity of CA, which affects less than 0.03% of the population (Salman, 2018), and the mobility restrictions faced by individuals with PD or CA. These challenges often result in prolonged study periods (e.g., 2 years) and small sample sizes, typically fewer than 15 participants (Breska and Ivry, 2018; Olivito et al., 2018; Wang et al., 2018). Moreover, many studies rely on participants from the same geographic area or family (McDougle et al., 2021), which leads to a lack of diversity in the sample and potential bias.

In addition, collecting sufficient data across multiple patient groups for sensitivity or specificity testing can be a significant challenge with traditional in-person testing methods. These limitations highlight the need for alternative approaches to data collection in neuropsychological studies. The challenges of traditional in-person methods have led to the rise of online methods in behavioral studies. Research shows that remote testing, including video telehealth approaches, is as reliable and valid as in-person testing (Casler et al., 2013; Chandler and Shapiro, 2016; Buhrmester et al., 2018; Bilder et al., 2020; Geddes et al., 2020; Marra et al., 2020; Saban and Ivry, 2021; Binoy et al., 2023).

Remote testing offers several advantages. For example, it makes research participation more convenient for individuals with neurological conditions by eliminating the need for travel (Barbosa et al., 2020). It also allows for rapid data collection and comprehensive assessments (Binoy et al., 2023), reaching a wider and more diverse pool of participants (Saban and Ivry, 2021; Binoy et al., 2023). However, online testing has its limitations. For example, it may be biased toward those with internet access and technological literacy, and home environment can vary (Hewitt et al., 2020).

In recent years, remote methods have been increasingly used to identify individuals with cognitive impairment. The Montreal Cognitive Assessment (MoCA; Nasreddine et al., 2005), a globally recognized test, is commonly used by healthcare providers and researchers to assess mild cognitive impairment (MCI). The MoCA has been employed to screen for mild cognitive impairment in PD and CA (Saban and Ivry, 2021), and to evaluate cognition (Butcher et al., 2017; Wu et al., 2017). However, a significant challenge lies in making remote evaluations of cognitive impairment, such as the MoCA, more accessible while ensuring their validity in both healthy and clinical populations.

Accordingly, with the growing use of technology, there has been a wide interest in the validity of administering the MoCA test remotely. A validated telephone version of the MoCA (T-MoCA) exists, which may be used when face-to-face (F2F) administration is not feasible (Klil-Drori et al., 2022). This telephone-based method could expand the recruitment pool to include individuals who need videoconferencing access. However, it is mainly useful for a simplified classification of patient cognitive status (Carlew et al., 2020). A preliminary study tested the T-MoCA on 21 PD participants, who also completed the traditional F2F MoCA. The study found only a modest correlation between the T-MoCA and traditional neuropsychological measures (verbal delayed recall = 0.35, trail making = 0.21, digit span = 0.55, Stroop interference trail = 0.39) (Benge and Kiselica, 2021). Another study tested an electronic version of the MoCA (eMoCA) via a touchscreen. The study compared the eMoCA to the regular paper version on a sample of 40 healthy older adults in the same session, finding a strong correlation (*r* = 0.68) between the two scores (Wallace et al., 2019). However, to our knowledge, the eMoCA has yet to be tested remotely on patients with PD or CA.

Several studies have investigated a video-conferencing version of the MoCA. A recent study compared MoCA scores obtained F2F with those obtained via video telehealth in a large sample of healthy English-speaking participants (Loring et al., 2023). The study found no differences between the two methods, supporting the validity of remote MoCA administration. Another study on English-speaking patients with mild-to-severe dementia found that the average MoCA score was not different in those tested remotely compared to those tested F2F, with an excellent intra-class coefficient reliability (ICC = 0.93) (Lindauer et al., 2017). Interestingly, a study amongst Japanese-speaking older adults found that the ICC for the MoCA was high overall but varied depending on the subgroup. The ICCs were lower in healthy controls (0.53) compared to those with mild cognitive impairment (MCI) (0.82) or dementia (0.82), and depended on disease severity (Iiboshi et al., 2020). Given these studies, it remains unclear whether online MoCA is valid in clinical populations.

A few studies have demonstrated the feasibility and efficiency of administering the MoCA on PD or CA participants through video conferencing. One study on a small sample of English-speaking PD patients (8) showed the feasibility of remotely administering the MoCA to these patients with movement disorders. However, this study did not compare the video conferencing patient data to F2F results (Abdolahi et al., 2016). In a pilot study on a small sample (*n* = 11) of English-speaking PD participants, participants completed the F2F MoCA and videoconferencing MoCA 1 week later (Stillerova et al., 2016). No differences were found between the two methods of administration; however, due to the small sample size, the validity of the videoconferencing MoCA remains to be tested. While most prior studies have small sample size, one study used a large database (*n* = 166) of PD participants. The researchers assessed patients via videoconferencing, which included the MoCA, showing feasibility of remote MoCA (Dorsey et al., 2015). Although this study had a large sample of PD participants, no comparison was made between patients and healthy participants. Online administration of the MoCA on participants with CA has been tested in a limited capacity by one study. In a pilot study administering a modified online version of the MoCA on a small sample of English-speaking CA participants (*n* = 18), no differences were found between online administration and previous in-person studies (Binoy et al., 2023).

As can be derived from reading previous literature, most of the studies that used the remote MoCA were conducted in English, with only one exception in Japanese. However, there is a notable over-reliance on English speakers in cognitive science (Blasi et al., 2022). English is the dominant language in the study of human cognition and behavior, and both the subjects of cognitive science studies and the researchers themselves are often English speakers. This reliance on English as the primary language of participants (and researchers) introduces a clear bias in the measurement of cognitive functions and hinder cognitive assessments (Blasi et al., 2022).

Online assessment allows for broader geographic reach and more diverse patient populations, supporting the generalizability of online testing in populations that do not consist solely of English speakers. The MoCA has been translated into 36 different languages, including Hebrew. While the in-person Hebrew version has been validated (Lifshitz et al., 2012), the remote version has yet to be validated in healthy or clinical populations.

To bridge the above-mentioned gaps, the current study aimed to assess the validity and generalizability of administering the MoCA online in two different languages (English and Hebrew) and across three populations: PD, CA, and healthy controls. We tested the convergent validity of the online MoCA by comparing our online data to in-person studies in all three groups, predicting no difference between the administration methods. The construct validity was also tested by comparing our online patient groups to healthy controls, hypothesizing similar patterns to previous in-person literature. Lastly, we examined the generalizability of online testing across different language-speaking populations: English and Hebrew.

## 2 Materials and methods

### 2.1 Participants

A total of 120 participants were evaluated. The participants responded to online advertisements (e.g., Facebook groups). For interested individuals, we followed-up with an email and a video call to describe the project in detail. Our initial recruitment email indicated that participation would require the ability to use a computer. Note that we ensured there were no video or audio issues before starting each session, so it would not interrupt the assessment. If there was any issue, we resolved it during the meeting or, in rare cases, rescheduled the session. For each participant, we obtained medical history, and we tested MCI using the MoCA (version 8.1). This protocol was approved by Tel Aviv University ethics committee and all participants provided informed consent.

See Table 1 for demographic information of all groups. Fifty percent of the participants (*n* = 60) were assessed via the English version, and the remaining participants via the Hebrew version (Lifshitz et al., 2012). All participants reported that they speak only one language, either Hebrew or English. For each language, we administered the MoCA to 20 participants in each group: Control, PD, and CA.

**TABLE 1:** Demographic summary of all groups (Mean [SD] (range)).

Group	Language	*n*	Age	Females	Education
Control	English	20	47.5 [12.7] (28–79)	11	16.1 [2.2] (11–20)
Control	Hebrew	20	51.2 [8.6] (34–66)	12	15.1 [3] (9–21)
CA	English	20	57.7 [12.7] (29–79)	10	16.8 [2] (12–20)
CA	Hebrew	20	52.5 [12.1] (34–88)	10	14.6 [3.4] (11–22)
PD	English	20	60.2 [7.6] (48–80)	9	16.9 [3.3] (12–27)
PD	Hebrew	20	64 [8] (48–77)	12	15.6 [2.1] (12–19)

The Hebrew-speaking CA group consisted of 17 individuals with a known genetic subtype of cerebellar ataxia (SCA3) and 3 with degenerative disorders of unknown etiology. Their mean duration since diagnosis was 6.1 (SD = 5.4) years and their SARA score was 12.1 (SD = 5.3). The English-speaking CA group consisted of 12 individuals with a known genetic subtype of CA (1 SCA1, 1 SCA28, 7 SCA3, 2 SCA5, 1 SCA6), and 8 with degenerative disorders of unknown etiology. Their mean duration since diagnosis was 5.5 (SD = 3.6) years and their SARA score was 13.3 (SD = 4.9). For the Hebrew and English-speaking PD group, we did not include individuals with surgical intervention (e.g., DBS), and all participants were tested while on their current medication regimen. The English-speaking PD group’s mean duration since diagnosis was 8 (SD = 4.9) years and their UPDRS score was 20.5 (SD = 5.3). The Hebrew-speaking PD group’s mean duration since diagnosis was 6.3 (SD = 4.5) years and their UPDRS score was 21.4 (SD = 12.6). All PD participants’ Hoehn and Yahr scores were below 4. The diagnosis of both patient groups was also based on self-report. Self-report assessment has evolved considerably in recent years, emerging as a robust and effective data collection method. Previous studies have found high concurrence rates between self-report and clinician-determined diagnosis (Kim et al., 2018; Winslow et al., 2018; Smolensky et al., 2020). The age across all groups ranged from 47.5 to 64 years, and MoCA scores did not change significantly within this age range (Rossetti et al., 2011; Freitas et al., 2012). The years of education of all groups ranged from 14.6 to 16.9 years, and it was found that variance above 12 years of education did not affect the MoCA score (Rossetti et al., 2011).

To calculate the required sample sizes, we conducted a power analysis (alpha = 0.05; power = 0.99) using effect sizes derived from five in-person studies that compared each patient group (PD or CA) and a neurotypical group in the MoCA test (PD: Hoops et al., 2009; Dalrymple-Alford et al., 2010; Hu et al., 2014; Kandiah et al., 2014; Biundo et al., 2016; *D* = −1.152: large effect size; CA: Tunc et al., 2019; Zhang et al., 2020; Chen et al., 2022; Schniepp et al., 2023; van Prooije et al., 2023; *D* = −1.265: large effect size). These analyses suggested a minimal sample size of 14 participants for each patient group (PD = 13.39; CA = 11.38). As such, the sample sizes of our groups (20) had sufficient power to detect group differences. To our knowledge, no previous studies compared the three groups on two different languages of the MoCA.

### 2.2 Online MoCA modification

Our online MoCA tests are in accordance with the official instructions that appear on the MoCA website. Changes were made to minimize deviations from standard F2F administration. Six MoCA items (visuospatial and naming) were presented using PowerPoint slides by the share screen Zoom option. For the trail-making test, participants were asked to say the number-letter sequence aloud (rather than drawing lines to connect the circle) according to the instructions provided on the official MoCA website for videoconferencing administration, which have been validated in a healthy control group (Loring et al., 2023). The copy cube stimulus slide contained “Cube copy” next to the figure to closely mimic the paper and pencil presentation. Similarly, the draw clock stimulus was presented on the screen during clock drawing. Participants were instructed to draw the cube and the clock on their own piece of paper and present their drawings in front of the camera. Following the clock drawing, participants were instructed to put the paper and writing utensils aside. Naming stimuli were presented individually on the screen. Orientation for place and city asked for the participant’s location.

## 3 Results

First, to assess the convergent validity of the online MoCA, we compared our online results to previous in-person studies (called “literature value”) using a one-sample *t*-test. For the literature value, we obtained the mean and standard deviation from relevant papers. Papers were selected based on their relevance (healthy, CA, and PD groups), with studies that administered the MoCA in-person and published within the last 15 years. The MoCA literature value used for the healthy control group comparison was taken from the original in-person MoCA validation study (Nasreddine et al., 2005). The literature value for the PD and CA groups were derived from five in-person studies for each group (PD: Hoops et al., 2009; Dalrymple-Alford et al., 2010; Hu et al., 2014; Kandiah et al., 2014; Biundo et al., 2016; CA: Tunc et al., 2019; Zhang et al., 2020; Chen et al., 2022; Schniepp et al., 2023; van Prooije et al., 2023).

The average MoCA score for our control group was 27.3, which is a value that falls within the normal range (>26). We did not find a significant difference from the literature value [μ = 27.4; *n* = 90, t(39) = 0.491, *p* = 0.626, effect size = 0.078]. Similarly, the online MoCA scores for the PD and CA groups were not significantly different from the literature values [PD: μ = 25.096, *n* = 523, t(39) = 1.770, *p* = 0.085, effect size = 0.280; CA: μ = 24.689, *n* = 195, t(39) = 0.846, *p* = 0.403, effect size = 0.134]. Given that the literature values were obtained in-person, these results show that our online approach produces typical results, supporting the convergent validity of the online approach in all groups.

**FIGURE 1:**
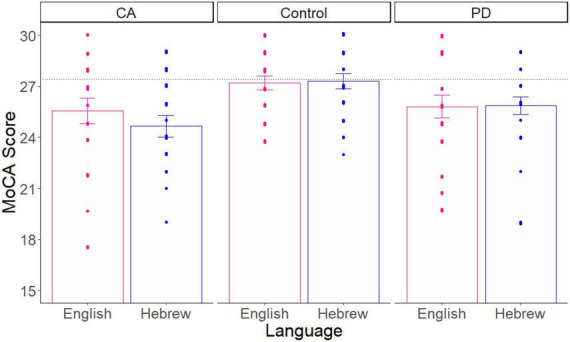
The average MoCA score as a function of group and language. The score for each participant is a dot. The black dotted line is the literature value of healthy control participants. Error bars = SE.

Second, since these patient groups typically show lower MoCA scores than healthy controls, we assessed the construct validity of the online MoCA by comparing our online neurotypical group to our patient groups. Third, to assess generalizability to other languages, we compared our Hebrew-speaking participants to our English-speaking participants. To achieve these last two goals, we carried out a two-way analysis of variance (ANOVA; in R software) with Group (Control, PD, CA) and Language (Hebrew, English) as the independent variables, and the MoCA score as the dependent measure. See Figure 1 for a comparison of the three groups and two languages (*n* = 60/Language).

As expected by previous literature, this analysis showed that there was a significant main effect of Group on the MoCA score [*F*_(2, 114)_ = 7.100, *p* = 0.001, effect size = 0.110]. Planned-comparison analyses revealed that the Control group performed significantly higher than the two patient groups, which did not differ significantly from each other [PD vs. Control: t(78) = 2.785, *p* = 0.006, effect size = 0.623; CA vs. controls: t(78) = 3.766, *p* = 0.0003, effect size = 0.842; PD vs. CA: t(78) = 1.139, *p* = 0.258, effect size = 0.255]. There was no significant main effect of Language [*F*_(1, 114)_ = 0.292, *p* = 0.590, effect size = 0.002]. Finally, we did not find a significant interaction effect between Group and Language [*F*_(2, 114)_ = 0.464, *p* = 0.630, effect size = 0.008]. These results demonstrate the construct validity and generalizability of the online MoCA test.

**FIGURE 2:**
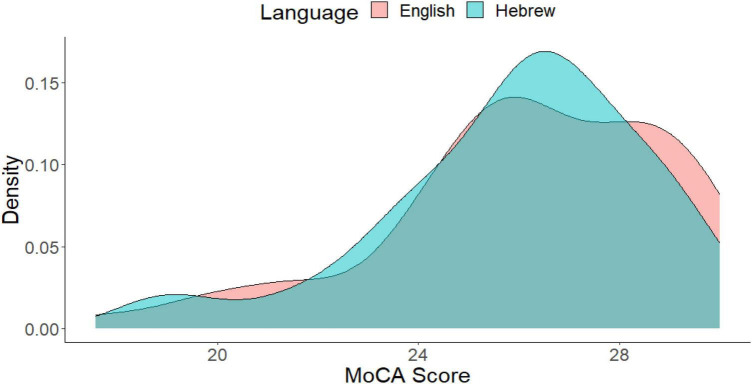
Histogram of the MoCA score as a function of **l**anguage. The degree of overlap between the two distributions is 91%.

We also examined the degree of overlap between the distributions of the two language versions of the MoCA, as shown in Figure 2. The overlap was defined as the intersection of the ranges of the two distributions, and we calculated the integral of the pointwise minimum of these densities over this range. The degree of overlap between the two distributions was found to be 91% (out of 100), indicating a high degree of overlap. This suggests that the two language distributions are similar to each other.

## 4 Discussion

Our study provides evidence supporting the equivalence of online MoCA testing with traditional in-person testing. We evaluated the validity of online MoCA administration in three groups: PD, CA, and healthy controls. The results indicate construct validity for online MoCA administration. As anticipated, the patient groups (PD and CA) scored lower than the healthy control group, reflecting the known cognitive impacts of these conditions. Interestingly, we observed no differences between the English and Hebrew versions of the test in each group. Our findings suggest that online MoCA testing is generalizable across two different language versions and can be valid across three populations.

Our current study has four primary limitations. Firstly, while our total sample size was 120 participants, each subgroup consisted of only 20 individuals. This limited sample size per subgroup restricts our ability to assess the specific items within the MoCA for each subgroup. This limited sample size per subgroup also restricts our ability to assess cognitive abilities within each specific subtype (e.g., SCA3 vs. SCA6). Future research could benefit from utilizing the remote MoCA to recruit larger sample sizes for each group. This would also allow for a more detailed analysis of individual MoCA items. Secondly, a more direct comparison between in-person and online administration could be achieved by conducting both types of assessments with the same participants from the PD and CA groups. This approach would provide a direct measure of validity by comparing these two testing methods. Third, conducting tests and experiments online may introduce an inherent selection bias, as participants are expected to have computer proficiency, potentially skewing the results in favor of technology-proficient populations. In our study, some participants found the attention and sentence repetition tasks more challenging due to internet connectivity issues that may obscure the audio. However, in general, participants provided positive feedback stating that they found the remote format to be more convenient, saving them the time and cost associated with traveling to an in-person testing site. Finally, there is also a potential selection bias in favor of participants with less disease severity who are more capable of participating in online studies. These considerations offer possible directions for future studies aiming to further our understanding of online cognitive assessments.

One interesting point of comparison is the eMoCA, which enables automated testing (Wallace et al., 2019). We propose that the videoconferencing MoCA has some advantages for PD and CA patients, as can be observed from our study. Videoconferencing MOCA is a simpler alternative as opposed to eMoCA which requires mailing a touchscreen tablet to participants or installing an application onto the participant’s device. Videoconferencing methods can also simplify the process for older participants who may not be as skilled with technology. Video conferencing allows researchers to maintain an adaptable human presence for participants to interact with while performing the assessment. Since videoconferencing involves a researcher who can mediate the computer interface, this allows a more accessible approach than the eMoCA, which requires independent work with a tablet and application. Additionally, the eMoCA is not available in all languages, including Hebrew. However, one limitation of videoconferencing methods compared to the eMoCA is that videoconferencing requires an administrator to be present, while the eMoCA is fully automated.

Despite these limitations, our study underscores the potential of the online MoCA in facilitating multinational data collection. The creation of a multinational database for patients with neurological conditions, especially rare conditions such as CA, can lead to a more representative and diverse sample size. Given that the MoCA is available in many languages, the remote version of the MoCA offers researchers an opportunity to gather more representative data across multiple language-speaking populations. This could help overcome major limitations in neuropsychological research (Saban and Ivry, 2021) related to language barriers and constraints imposed by the neurological conditions being tested.

We collected data from 120 participants in a 1-month period. Thus, the online approach is not only valid but much more efficient in terms of data collection. Our sample included individuals who are currently residing in more than 20 USA states, such as New York and California, and different cities in Israel, such as Jerusalem and Tel Aviv. Accordingly, our online sample is much more geographically diverse than a typical laboratory-based study. Recruitment across countries and different populations is critical for diversity and, potentially, better representation of the population. Our remote videoconferencing MoCA shows promise in the avenue of telemedicine, which is especially useful for early detection and screening of neurodegenerative conditions that require long-term repeated evaluation of symptoms. This would improve accessibility for rural and underserved populations, as well as those with mobility restrictions.

Remote testing presents an encouraging avenue for broadening the scope and depth of neuropsychological research. It opens possibilities for investigating interactions between other areas of function, such as motor abilities, and comparing conditions like PD and CA with other neurological conditions, such as Huntington’s disease. There is a need for future studies to develop new strategies to validate remote diagnostic tests, such as the SARA and the UPDRS.

Our promising results underscore the potential use of technological advancements to revolutionize both research and clinical communities. Establishing the validity of online cognitive assessment is particularly vital in the era of tailored therapy and biomarker development. Online cognitive batteries using large and diverse cohorts can allow researchers to profile cognitive abilities more accurately in different pathologies. The increased efficiency in collecting data and diversity in sample sizes resulting from binational efforts could significantly enhance our understanding of PD and CA. The impact of technological advancements could lead to improved diagnosis, treatment, and care for individuals affected by these conditions. It is our hope that neuropsychological assessment developments will continue to evolve in tandem with technological capabilities, ultimately benefiting patients worldwide.

## Data availability statement

The original contributions presented in the study are included in the article/supplementary material, further inquiries can be directed to the corresponding author.

## Ethics statement

The studies involving humans were approved by the Tel Aviv University Center for Accessible Neuropsychology. The studies were conducted in accordance with the local legislation and institutional requirements. The participants provided their written informed consent to participate in this study.

## Author contributions

SB: Writing – original draft, Writing – review and editing. LM-K: Writing – review and editing. PP: Writing – review and editing. WS: Conceptualization, Data curation, Formal analysis, Funding acquisition, Investigation, Supervision, Validation, Writing – original draft, Writing – review and editing.
