# Corrigendum: Remote assessment of cognition in Parkinson's disease and cerebellar ataxia: the MoCA test in English and Hebrew

**DOI:** 10.3389/fnhum.2024.1401098

**Published:** 2024-04-04

**Authors:** Sharon Binoy, Leila Montaser-Kouhsari, Penina Ponger, William Saban

**Affiliations:** ^1^Center for Accessible Neuropsychology and Sagol School of Neuroscience, Tel Aviv University, Tel Aviv-Yafo, Israel; ^2^Department of Occupational Therapy, Faculty of Medicine, Tel Aviv University, Tel Aviv-Yafo, Israel; ^3^Loyola Stritch School of Medicine, Chicago, IL, United States; ^4^Department of Neurology and Neurological Sciences, Stanford University, Stanford, CA, United States; ^5^Movement Disorders Division, Department of Neurology, Tel Aviv Sourasky Medical Center, Tel Aviv-Yafo, Israel

**Keywords:** online, neuropsychological testing, MOCA, Parkinson's, ataxia, basal ganglia, cerebellum, Hebrew

In the published article there was an error in the **author list**. The second author's name was incorrectly spelt “Leila Monstaser-Kouhsari”. This should have been written as “Leila Montaser-Kouhsari”.

In the published article there was an error in the **citation section**. The second author's name citation was incorrectly spelt “Monstaser-Kouhsari L”. This should have been written as “Montaser-Kouhsari L”.

In the published article there was an error in the **copyright section**. The second author's surname was incorrectly spelt “Monstaser-Kouhsari”. This should have been written as “Montaser-Kouhsari”.

The authors apologize for these errors and state that this does not change the scientific conclusions of the article in any way. The original article has been updated.

